# Supplementary material for: Mu Opioid Receptor 1 (MOR-1) Expression in Colorectal Cancer and Oncological Long-Term Outcomes: A Five-Year Retrospective Longitudinal Cohort Study
Source: Cancers (Basel). 2020 Jan 5;12(1):134. doi: 10.3390/cancers12010134 (PMC7016725; doi:10.3390/cancers12010134)
Supplement: Supplementary file 1 [file cancers-12-00134-s001.pdf]

Supplementary Materials: Mu Opioid Receptor 1 (MOR-1) Expression in Colorectal Cancer and Oncological Long-Term Outcomes: A Five-Year Retrospective Longitudinal Cohort Study

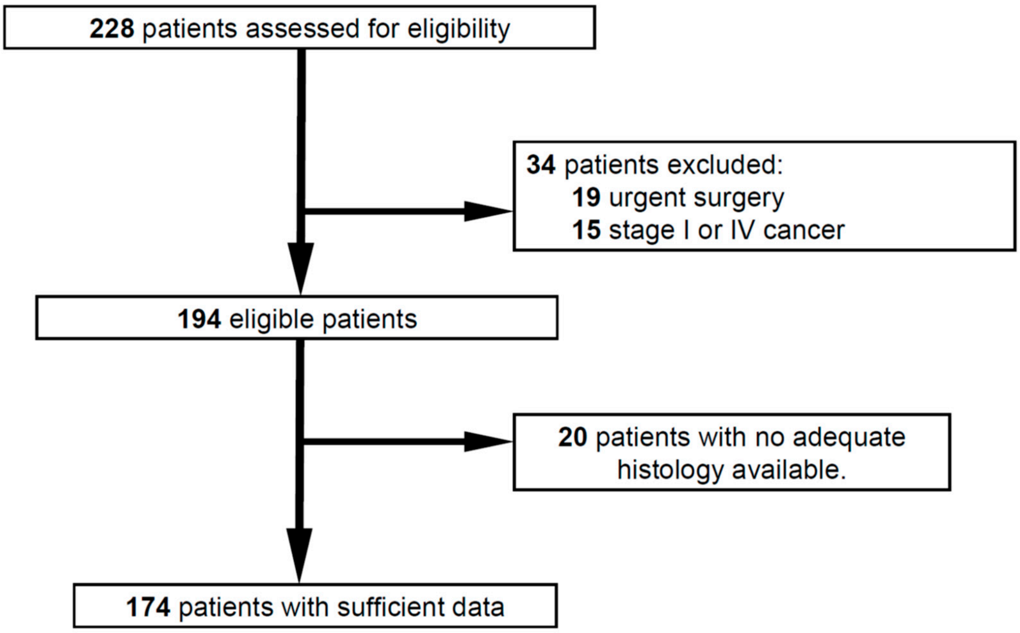

Figure S1. Patients' flowchart.

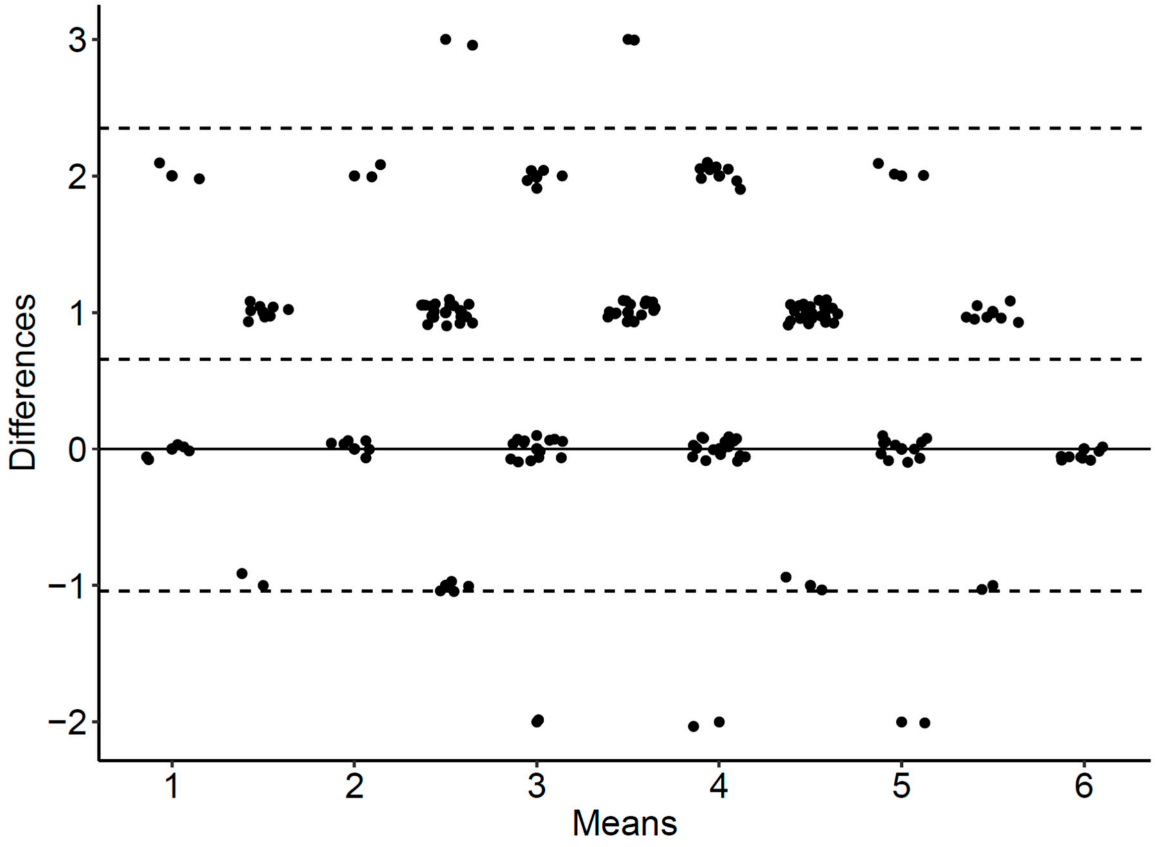

Figure S2. Bland–Altman plot of the agreement between two independent readings.

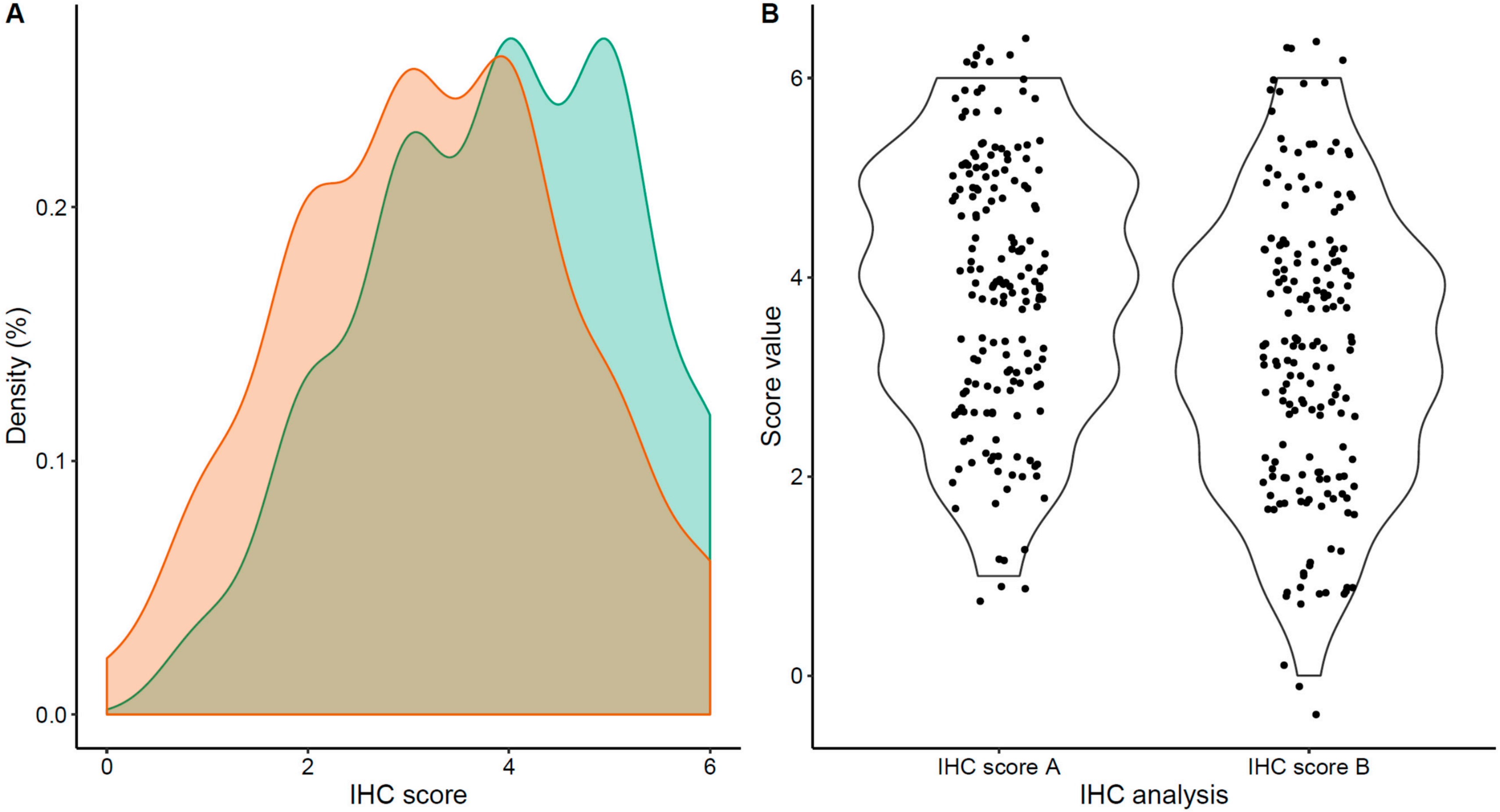

Figure S3. MOR-1 expression scoring: (**a**) Probability density plot of MOR-1 score, Green: IHC score A, Orange: IHC score B; (**b**) Scatterplot and Violin plot of score distribution by analysis.

Table S1. Association between Mu–opioid receptor expression score and oncological features.

Characteristics	N	Correlation	Lower– Upper 95% CI	P value
**Ca 19–9** (U·mL^{-1})	124	−0.07*	−0.26 – 0.12	0.448
**CEA** (U·mL^{-1})	163	0.05*	−0.09 – 0.22	0.497
**Number of positive lymph nodes**	174	0.15*	0.003 – 0.29	0.044
**Duke stage**		0.19**	0 – 0.31	0.119
A	2			
B	78			
C	71			
D	2			
**Cancer stage**		0.15**	0 – 0.28	0.053
II	96			
III	78			
**Resection margin**		0.008**	0 – 0.13	0.926
R+	25			
R0	110			
**Tumoral tissue differentiation**		0.06**	0 – 0.18	0.717
Poor/Undifferentiated	20			
Moderately differentiated	132			
Well differentiated	17			

Ca 19-9, Gastrointestinal Cancer Antigen; *CEA*, Carcinoembryonic antigen; *R+*, positive margin; *R0*, negative margin; *CI*, Confidence interval. * Spearman correlation coefficient. ** square root of ANOVA η^2

Table S2. Multivariable logistic regression model for complications at 28 postoperative days.

	Odds Ratio	Lower – Upper 95%CI	*P* value
**MOR expression tumor tissues**	0.711	0.340 – 1.310	0.305
**MOR expression non–tumour tissue**	0.555	0.188 – 1.400	0.237
**First postoperative 96 hours opioid dose** (morphine equivalents)	1.012	0.989 – 1.039	0.342
**First postoperative 96 hours transfusion** (yes)	0.819	0.123 – 5.055	0.828
**Gender** (Female)	0.391	0.052 – 2.100	0.299
**ASA** (Reference category = 1)			
2	-0.147	-2.438 – 1.510	0.996
≥3	0.224	-2.066 – 2.395	0.995
**Surgery duration** (minutes)	1.004	0.991 – 1.018	0.539
**Arterial Hypertension** (yes)	4.383	0.717 – 42.894	0.141
**Number of affected lymph nodes**	3.610	1.546 – 10.789	0.008
**Serum total proteins** (g·dL^{-1})	1.024	0.832 – 1.920	0.879
**Smoker** (yes)	0.779	0.026 – 9.585	0.86
**Age** (years)	1.007	0.921 – 1.107	0.878

MOR, Mu–opioid receptor; *ASA,* American Society of Anesthesiology; *CI*, Confidence Interval.

Table S3. Study's REMARK profile.

A. Patients, treatment and variables	
Study and marker	Remarks
Marker	MOR–1: Immunohistochemical cell staining assessed on an ordinal scale from 0 to 6 (see Table 3) in tumour tissue
Further variables	v1= Gender
	v2= Anaesthetic agent (intravenous vs. halogenated)
	v3= Complication in the first 28 postoperative days (yes/no)
	v4= Amount of opioid drugs administered in the first 96 postoperative hours (in morphine equivalents)
	v5= Blood transfusion in the first 96 postoperative hours
	v6= Epidural analgesia (yes/no) v7= neo–adjuvant radiotherapy
	v8 = Neo–adjuvant chemotherapy
	v9 = Adjuvant chemotherapy
	v10= Adjuvant radiotherapy
	v11= ASA risk score
	v12 = Duration of surgery
	v13= Preoperative Haemoglobin value
	v14= Stage III cancer (%)
	v15 = Reintervention
	v16= MOR–1 expression in non–tumor tissue
	v17= Carcinoembryonic antigen value at diagnosis (log scale)
	v18= Number of positive lymph nodes (log scale)
	v19= Age
	v20 = Arterial Hypertension
	v21= Diabetes
	v23= Smoker

	v24= Preoperative Plasma Total Protein	
**Patients**	N	Remarks
Assessed for eligibility	228	*Disease*: Colorectal cancer stage II and III.<br>*Patients:* Surgery 2010 to 2013, Hospital Universitario la Fe, Valencia, Spain.<br>*Sample:* Archived specimens available.
Excluded	54	
Included	174	
With outcome events	174	

B. Statistical analyses of outcomes

**Analysis**	**Patients**	**Events**	**Variable considered**	**Results/Remarks**
A1: MOR–1 expression in tumour and non–tumour tissue	174	–	MOR–1 , v18	Text (Page 5,Line 76)
A2: DFS Univariable	174	40	MOR–1	Text (Page 4,Line 88)
A3: DFS Multivariable. Variable selection process	109	25	MOR–1,v1 to v24	Elastic net with hyperparameters estimated by cross validation $\alpha = 0.8$ $\lambda = 0.1$
A4a: Adjusted Effect of MOR –1on DFS	135	30	MOR–1,v5,v11,v13,v17 to v19	Table 2
A4b: Adjusted Effect of MOR–1 on DFS with missing values imputation	174	40	MOR–1,v5,v11,v13,v17 to v19	Table 2
A5: OS Univariable	174	40	MOR–1	Text (Page 4, Line 90)
A6: OS Multivariable. Variable selection process	109	26	MOR–1,v1 to v24	Elastic net with hyperparameters estimated by cross validation $\alpha = 0.8$ $\lambda = 0.08$
A7: Adjusted Effect of MOR –1 on OS	135	29	MOR–1,v5,v11,v13,v17 to v19	Table 2
A7b: Adjusted Effect of MOR–1 on OS with missing values imputation	174	40	MOR–1,v5,v11,v13,v17 to v19	Table 2

A8: Complications Univariable	174	32	MOR–1	Text (Page 6, Line 113)
A9: Complications Multivariable	87	11	MOR–1, v1 to v24 omitting v15 which is a complication	Elastic net with hyperparameters estimated by cross validation $\alpha = 0.8$ $\lambda = 0.08$
A10: Adjusted Effect of MOR–1 on complications	74	16	MOR–1, v1,v4,v5,v11,v12,v15, v18,v20,v23,v24	Table S2

Table S4. Multivariable Cox regression model for disease free survival and overall survival at 5 years follow–up. Sensitivity analysis with chemotherapy and tumor stage as covariables.

	**Complete cases model**		
**Disease free survival model**	**N = 132 Events = 30**		
	Hazard Ratio	Lower – Upper 95%CI	P –value
**MOR expression**	0.786	0.597 – 1.036	0.087
**First postoperative 96 hours transfusion (yes)**	1.069	0.418 –2.73	0.889
**ASA** (Reference category = 1)			
2	0.587	0.122 –2.816	0.506
3	0.855	0.176 – 4.147	0.845
4	1.126	0.132– 9.603	0.913
**Preoperative Hemoglobin** ($g·dL^{-1}$)	1.050	0.850 –1.297	0.649
**Number of affected lymph nodes**	1.871	1.177 – 2.974	0.008
**CEA at diagnosis** ($U·mL^{-1}$)	1.200	0.830 –1.735	0.333
**Age** (years)	1.021	0.978 – 1.066	0.349
**Stage III** (yes)	0.793	0.287 – 2.194	0.655
**Adjuvant chemotherapy** (yes)	1.304	0.528 – 3.22	0.565
**Overall survival model**	N = 132 Events = 29		
**MOR expression**	0.982	0.754 – 1.278	0.891
**First postoperative 96 hours transfusion (yes)**	1.555	0.649 – 3.726	0.322
**ASA score** (Reference category = 1)			
2	0.993	0.119 – 8.272	0.995
3	1.969	0.241 – 16.092	0.527
4	2.526	0.215 – 29.625	0.461
**Preoperative Hemoglobin** ($g·dL^{-1}$)	0.916	0.734 – 1.143	0.435
**Number of affected lymph nodes**	1.340	0.800 – 2.245	0.265
**CEA at diagnosis** ($U·mL^{-1}$)	1.639	1.156 – 2.324	0.006
**Age** (years)	1.036	0.992– 1.082	0.114
**Stage III** (yes)	1.236	0.426– 3.588	0.696
**Adjuvant chemotherapy** (yes)	1.380	0.567– 3.363	0.478

MOR–1 expression is introduced in both models as a 0 to 6 ordinal variable. The effect estimate is thus to be interpreted as the difference in hazard in the monitored time period when MOR–1 expression increases one level.

© 2019 by the authors. Submitted for possible open access publication under the terms and conditions of the Creative Commons Attribution (CC BY) license (http://creativecommons.org/licenses/by/4.0/).
